# Function of miR-24 and miR-27 in Pediatric Patients With Idiopathic Nephrotic Syndrome

**DOI:** 10.3389/fped.2021.651544

**Published:** 2021-04-21

**Authors:** Fen-fen Ni, Guang-lei Liu, Shi-lei Jia, Ran-ran Chen, Li-bing Liu, Cheng-rong Li, Jun Yang, Xiao-Jie Gao

**Affiliations:** ^1^Department of Nephrology, Shenzhen Children's Hospital, Shenzhen, China; ^2^The Fifth Affiliated (Zhuhai) Hospital of Zunyi Medical University, Zhuhai, China; ^3^Department of Immunology, Shenzhen Children's Hospital, Shenzhen, China

**Keywords:** idiopathic nephrotic syndrome, miR-24, Th2 cells, IL-4, IL-13, miR-27

## Abstract

**Purpose:** We investigated the pathogenesis of idiopathic nephrotic syndrome (INS) by measuring the effects two specific miRNAs on Th2 cells in children with this disease.

**Methods:** After informed consent, we enrolled 20 children with active INS before steroid initiation, 20 children with INS in remission after steroid therapy, and 20 age-matched healthy controls. Flow cytometry was used to measure the levels of Th2 cells and a cytometric bead array was used to measure the levels of IgE, interleukin (IL)−4, and IL-13. RT-PCR was used to measure the levels of miR-24 and miR-27 in CD4^+^TCD25^−^ cells. PBMCs were isolated using Ficoll density gradient centrifugation, and transfected with different mimic or inhibitor miRNAs. RT-PCR was used to measure the expression of different RNAs, and flow cytometry was used to determine the percentage of Th2 cells.

**Results:** Relative to healthy controls, children with active INS had higher percentages of Th2 cells (*P* < 0.05), but there was no significant difference in controls and children in remission. The plasma levels of IgE, IL-4, and IL-13 were significantly increased in children with active INS (*P* < 0.05). There were lower levels of miR-24 and miR-27 in children with active non-atopic INS (*P* < 0.05). Transfection experiments indicated that upregulation of each miRNA decreased the percentage of Th2 cells and the level of IL-4 (*P* < 0.05), and down-regulation of each miRNA had the opposite effects (*P* < 0.05).

**Conclusion:** Children with active INS, with or without atopy, had higher levels of IgE, possibly related to their higher levels of IL-13 and IL-4 due to a drift toward Th2 cells. miR-24 and miR-27 suppressed the expression of Th2 cells and have a critical function regulating Th2 cell expression in INS.

## Introduction

Idiopathic nephrotic syndrome (INS) is the most common renal disease in children, and a main cause of chronic renal failure in children from China (1). The clinical manifestations of INS are proteinuria, low level of plasma albumin, hyperlipidemia, and edema. The prevalence is greatest in preschool children who are 3–5 years-old. About 80–90% of patients with kidney disease who are under 10 years-old have minimal change disease (MCD), a common cause of INS (2). However, the specific etiology and mechanism of INS in children remain unclear. Previous studies showed that INS is associated with immune system dysfunctions, including humoral immune disorders, T cell subset dysfunction, and abnormal secretion of cytokines, especially due to T cell dysfunction (3–8).

T helper type 2 (Th2) cells, which mainly produce IL-4, IL-5, and IL-13, play a major role in responses to parasitic infections and allergic inflammatory diseases (9–11). A seminal 1959 study reported an association of pollen sensitivity with seasonal proteinuria, and that about 30% of children with INS had atopic manifestations, such as allergic rhinitis and idiopathic dermatitis (12). Subsequent studies confirmed elevated levels of immunoglobulin E (IgE) during the active phase of INS (13–15), but it is unknown whether the increased levels of IgE in these children are pathogenic or coincidental.

Previous researchers proposed a “two hit” hypothesis for the pathogenesis of INS. The “first hit” occurs when microbial products, allergens, or T-cell cytokines (such as IL-13) damage the glomeruli, resulting in the overexpression of CD80 by podocytes and temporary proteinuria (16–18). In normal settings, regulatory cytokines produced by T regulatory cells (Tregs) terminate the CD80 overexpression, so that the proteinuria is transient and mild (18–20). However, a “second hit” occurs when MCD is present, and this leads to a failure to block CD80 expression by podocytes due to a disruption of autoregulatory responses in Tregs or even in the podocytes themselves. After this second hit, CD80 expression remains continuously elevated, leading to nephrotic syndrome (20). Th2 related cytokines such as IL-13, which stimulates IgE-mediated responses, can promote proteinuria in patients with MCD because it can directly induce CD80 expression in podocytes (13–15, 18). Recent studies found increased levels of IL-13 and IL-4 during the active phase of INS, and that IL-13 functions in the pathogenesis of kidney disease (13–15, 18), but the mechanisms responsible for the increased level of IL-13 are still unclear. Several studies also reported that Th2 cells were over-active in INS (8, 18, 21, 22), but the mechanisms leading to over-activation of these cells are also unknown. Studies of these topics may help to elucidate the role of altered immune responses in the pathogenesis of INS.

MicroRNAs (miRNAs) are short (20–23 nucleotides) non-coding RNAs that can alter the expression of targeted genes (23, 24). In particular, a specific miRNA binds to the 3′-untranslated region (3′-UTR) of its target mRNA, and this is followed by inhibition of translation or increased mRNA degradation (23, 24). Initially, most studies of miRNAs were in the field of oncology, but recent studies have also examined their role in kidney disease (25, 26). For example, several studies showed that multiple miRNAs can inhibit the differentiation and function of Th2 cells (27–29). In 2016, researchers studying allergies found that miR-24 and miR-27 also inhibited the function of Th2 by inhibiting the production of cytokines such as IL-4 (28, 29). However, the effects of these two miRNAs on Th2 expression in patients with active INS, and their specific mechanisms need further study.

Atopic patients have abnormally increased proportions Th2 cells and related factors (9–11). However, it is not clear whether these alterations in children with INS are related to atopy and the pathogenesis of nephropathy. Thus, we systematically measured the dynamics of multiple cytokines, miR-24, and miR-27 in the regulation of Th2 cell differentiation during the active and remission phases of INS, and measured changes in the number and function of Th2 cells isolated from children with non-atopic INS under conditions of altered miRNA expression. Our general purpose was to clarify the role of immune system alterations in the pathogenesis of INS, and to identify potential therapeutic targets and new ideas for the treatment of kidney disease.

## Patients and Methods

### Subjects

Forty children with INS (23 males and 17 females; median age: 38 months; age range: 22–92 months) were enrolled as patients and were divided into two groups: before steroid initiation (11 males and nine females; median age: 35 months; age range: 22–84 months) and after steroid therapy (12 males and eight females; median age: 41 months; age range: 26–92 months). There were also 20 healthy volunteers of similar age (11 males and nine females; median age: 31.3 months; age range: 25–105 months) enrolled as controls (Ctrl) who visited the hospital for physical exams.

All patients were examined at Shenzhen Children's Hospital from September 2015 to October 2016. The inclusion criteria were diagnosis of INS based on the 2010 criteria determined using evidence-based diagnosis and treatment guidelines for common kidney diseases in children from China (30). The 40 children were divided into four groups: an active phase (first-onset) group with atopic constitution (AA, *n* = 6), an active phase group with non-atopic constitution (ANA, *n* = 14), a remission group with atopic constitution (ReA, *n* = 6), and a remission group with non-atopic constitution (ReNA, *n* = 14). Atopy was diagnosed based on family history, the presence of relevant symptoms (asthma, recurrent urticaria, eczema, and allergic rhinitis), and elevated serum IgE concentration. The results of the skin-prick tests were positive for all atopic children. Prednisone therapy was initiated at a dose of 2 mg/kg/day. All patients were steroid-sensitive, had negative test results for urinary protein within 4 weeks of treatment, completed the treatment protocol, and received no other immunosuppressants. In addition, none of the patients had a secondary kidney disease (secondary nephrotic syndrome, nephrotic syndrome, congenital kidney disease, etc.) and none had other systemic visceral syndromes. Blood samples were collected for analysis before steroid initiation in the active NS group and 4 weeks after steroid discontinuation in the remission group.

All parents or legal guardians provided informed consent prior to study enrollment, and the study was performed after approval by the local Medical Ethics Committee.

### Blood Samples

Venous blood was collected from patients and healthy controls in EDTA tubes. Then, Ficoll density gradient centrifugation was used to isolate peripheral blood mononuclear cells (PBMCs) for analysis by flow cytometry. Plasma samples were collected after centrifugation and were frozen (at −80°C) prior to using the cytometric bead array (CBA; kit no. 11363D, Dynal, Invitrogen, USA) for isolation of CD4^+^CD25^−^ T cells). Cell purity was based on a threshold of 97% from flow cytometry. Cell activity (determined using the trypan blue exclusion assay) was based on a threshold of 95%.

### Extraction of Total RNA Extraction and Synthesis of cDNA

Total RNA (including miRNAs) samples were isolated from CD4^+^CD25^−^ T cells using the miRNeasy Mini Kit (Qiagen, Germany) according to the manufacturer's instructions. Following confirmation of purity (average OD_260nm_/OD_280nm_ = 1.98), cDNA was synthesized using oligo-dT primers and RevertAid™ H Minus reverse transcriptase (Fermentas, Lithuania). The miScript II RT kit (Qiagen) was used for synthesis of miRNA cDNAs. Negative controls (no first-strand synthesis) were synthesized using reverse transcription without reverse transcriptase.

### LightCycler Real-Time PCR

The level of IL-4 was determined using real-time PCR with the Quantitect™ SYBR green PCR Kit (Takara, Japan) and a LightCycler^®^ 2.0 (Roche Molecular Biochemicals, Switzerland). The primers were: 5′-TCATTTTCCCTCGGTTTCAG-3′ (forward) and 5′- ATAGGTGTCGATTTGCAGTG-3′ (reverse). Real-time PCR with the miScript SYBR Green PCR Kit (Qiagen) and the LightCycler^®^ 2.0 were used to quantitate the levels of miR-24 and miR-27. The primers were synthesized from recommendations in the miRNA miScript Primer Assay (Qiagen). The second derivative maximum method was used to identify the crossing point (Cp) with LightCycler software version 3.5.30 (Roche Molecular Biochemicals). After normalization using Relative Quantification Software version 1.0 (Roche Molecular Biochemicals), the levels of the PCR products were presented relative to those of *GAPDH* (target genes) or *U6* (miRNAs).

### Flow Cytometry Analysis of Th2 Cells

To measure expression of cytoplasmic markers of Th2 cells, cells were cultured in a 37°C/5% CO_2_ incubator, with ionomycin (250 ng/mL; SigmaAldrich), PMA (25 ng/mL; Sigma-Aldrich), and monensin (20 ng/mL; eBio-science, San Diego, CA, USA) added as stimulation for 24 h. Then, cells were stained for different markers, and were fixed and permeabilized using the Cytofix/Cytoperm kit (eBioscience). The cells were stained with specific antibodies (or corresponding isotype-matched controls) and then analyzed using a FACS Canto II flow cytometer (BD Biosciences, Mississauga, ON, Canada). Staining with the fixable viability stain 450 (FVS450, BD Biosciences) was used to determine viability. Validated antibodies (CD3-eFour450A, CD8-FITC, and IL-4-PE) were purchased from eBioscience.

### CBA Detection of Plasma IgE, IL-4, and IL-13

A CBA kit (e Bioscience) was used to measure the plasma levels of IgE, IL-4, and IL-13. Each sample was measured twice.

### Cell Transfection and Culture

The roles of miR-24 and miR-27 in Th2 cells were examined using transfection experiments in PBMCs. First, PBMCs were isolated from healthy controls or patients using Ficoll density gradient centrifugation. Then mimics (Ctrl-m, miR-24-m, or miR-27-m) or inhibitors (Ctrl-i, miR-24-i, or miR-27-i) from RiboBio (Guangzhou, China) were transfected into the PBMCs using the riboFECT CP Transfection Kit (Guangzhou, China). Cells were cultured in RPMI-1640 medium (Gibco, CA, USA) that was supplemented with 15% fetal calf serum (Gibco, CA, USA) and maintained at 37°C/5% CO_2_ in 24-well plates (3 ×10^6^ cells per mL). Cells were then harvested for flow cytometry and RT-PCR analyses.

### Statistical Analysis

Statistical analyses were performed using SPSS software for Windows version 13.0 (SPSS Inc., USA). Data are expressed as means ± standard deviations. For comparisons of multiple groups, a one-way analysis of variance was used. For comparison of two groups, Student's *t-*test was used. *P*-values below 0.05 were considered significant.

## Results

### Patients With Atopic and Non-atopic INS Have Increased Serum IgE Levels

**Figure 1:**
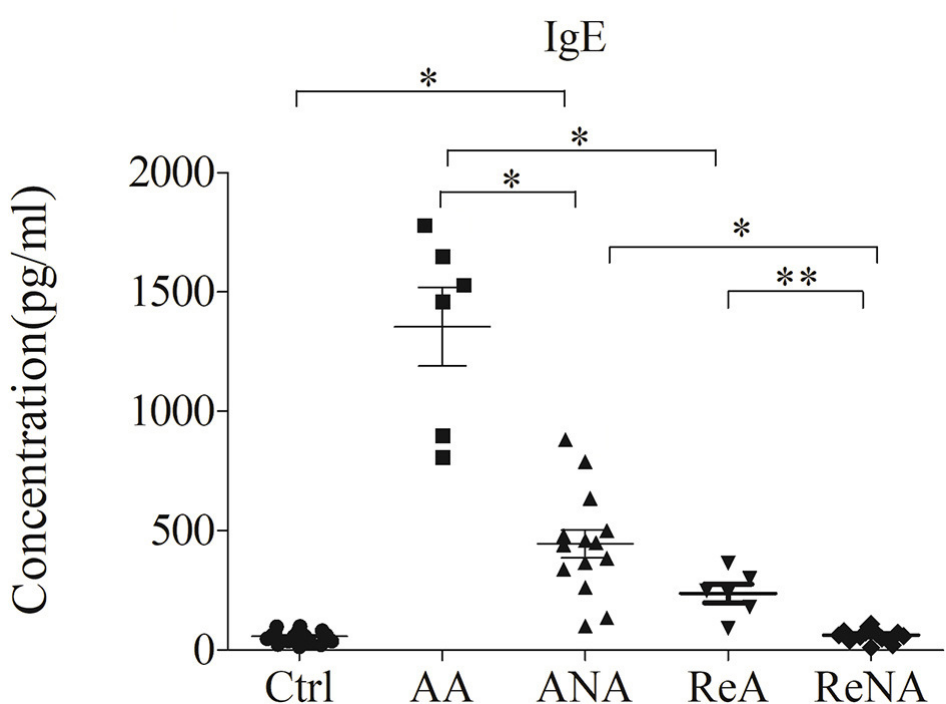
Children with atopic and non-atopic INS have increased serum levels of IgE. Here and below: all data are shown as means ± SDs; ^*^P < 0.05 and ^**^P > 0.05; and abbreviations are healthy control (Ctrl, *n* = 20), active phase atopic (AA, *n* = 6), active phase non-atopic (ANA, *n* = 14), remission phase atopic (ReA, *n* = 6), and remission phase non-atopic (ReNA, *n* = 14).

We first measured the plasma levels of IgE in the five different groups using a CBA (Figure 1). Analysis of IgE concentration indicated significantly greater levels in the AA group (1350.67 ± 837.39 IU/mL, *P* < 0.05) and the ANA group (441.01 ± 357.45 IU/mL, *P* < 0.05) than in the Ctrl group (57.76 ± 48.25 IU/mL). In addition, the AA group had higher levels of IgE than the other groups (all *P* < 0.05). Compared with the ANA group, the Ctrl, ReNA (62.97 ± 27.31 IU/mL), and ReA (237.75 ± 95.15 IU/mL) groups had significantly reduced levels of IgE (all *P* < 0.05). However, the ReA and ReNA groups had no significant difference (*P* > 0.05).

### Patients With Active Phase INS Have Over-Expression of Th2 Cells and Associated Cytokines

**Figure 2:**
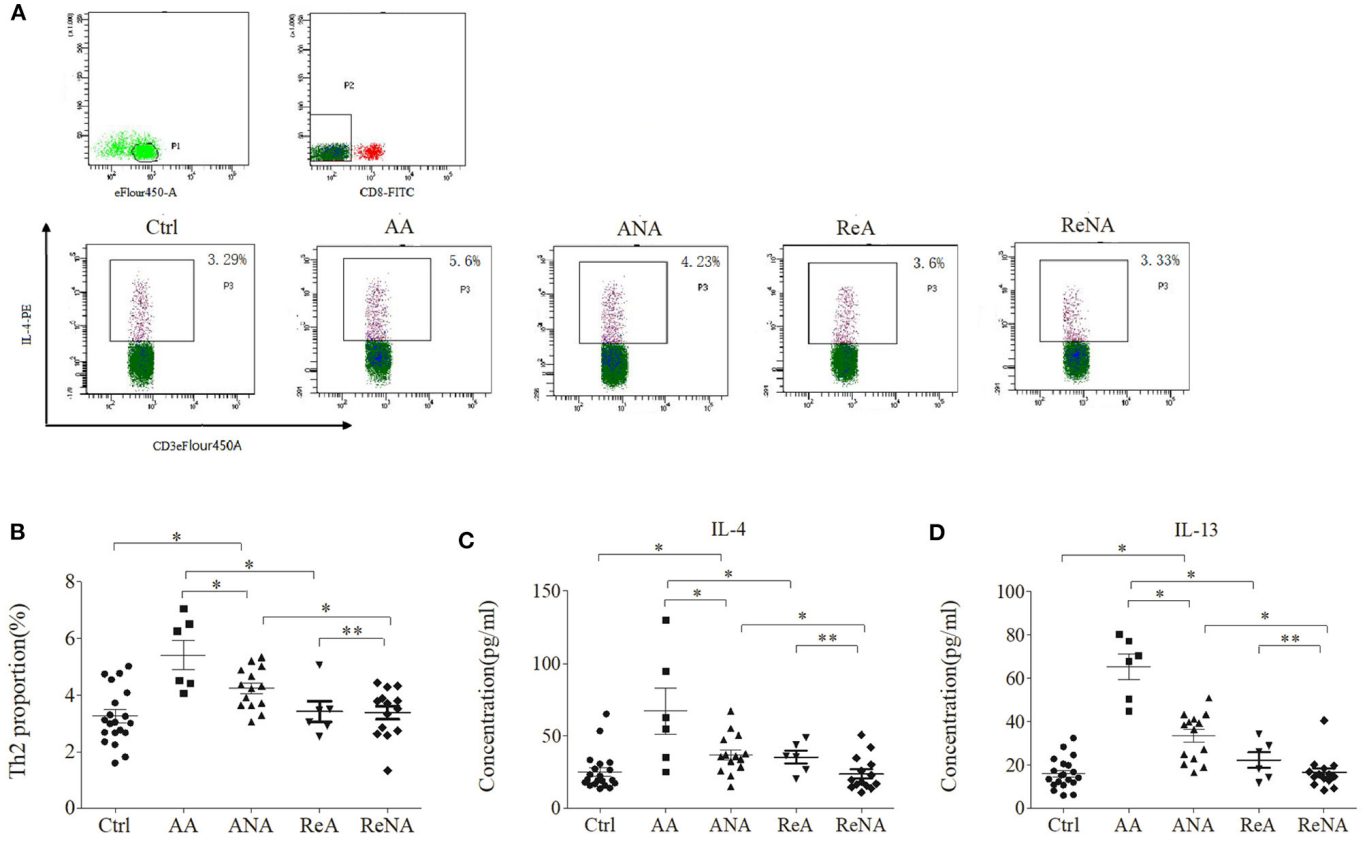
Children with INS have increased levels of Th2 cells and Th2-related cytokines. **(A)** Flow cytometric analysis of Th2 cells. **(B)** Percentages of Th2 cells. **(C)** Plasma concentrations of IL-4. **(D)** Plasma concentrations of IL-13. ^*^P < 0.05 and ^**^P > 0.05.

We quantified the Tregs from whole blood samples in the five groups using flow cytometry (Figures 2A,B). The percentage of peripheral Th2 cells in the AA group (5.60% ± 1.21) and the ANA group (4.23% ± 0.92) were significantly greater than in the Ctrl group (3.29% ± 1.02, both *P* < 0.05). As with IgE, there was no significant difference between the ReA (3.60% ± 0.78) and ReNA (3.40% ± 0.64) groups (*P* > 0.05). Also as with IgE, the AA group had a higher percentage of Th2 cells than all other groups (all *P* < 0.05).

We then measured the plasma levels of IL-4 and IL-13 in all five groups using CBA (Figures 2C,D). Relative to the control group, the AA group had higher levels of IL-4 (66.66 pg/mL ± 43.85 vs. 23.29 pg/mL ± 13.91, *P* < 0.05) and IL-13 (65.15 pg/mL ± 16.50 vs. 15.66 pg/mL ± 8.16, *P* < 0.05). Also relative to the control group, the ANA group had higher levels of IL-4 (37.04 pg/mL ± 17.39 vs. 23.29 pg/mL ± 13.91, *P* < 0.05) and IL-13 (33.15 pg/mL ± 14.09 vs. 15.66 pg/mL ± 8.16, *P* < 0.05). However, there was no significant difference between the ReA and ReNA groups in these cytokines (IL-4: 35.91 pg/mL ± 15.75 vs. 23.73 pg/mL ± 11.71, *P* > 0.05; IL-13: 22.33 pg/mL ± 8.87 vs. 16.53 pg/mL ± 7.76, *P* > 0.05). Compared with the AA group, all other groups had significantly decreased levels of both cytokines (all *P* < 0.05).

### Patients With Non-atopic INS Have Altered Expression of miR-24 and miR-27

**Figure 3:**
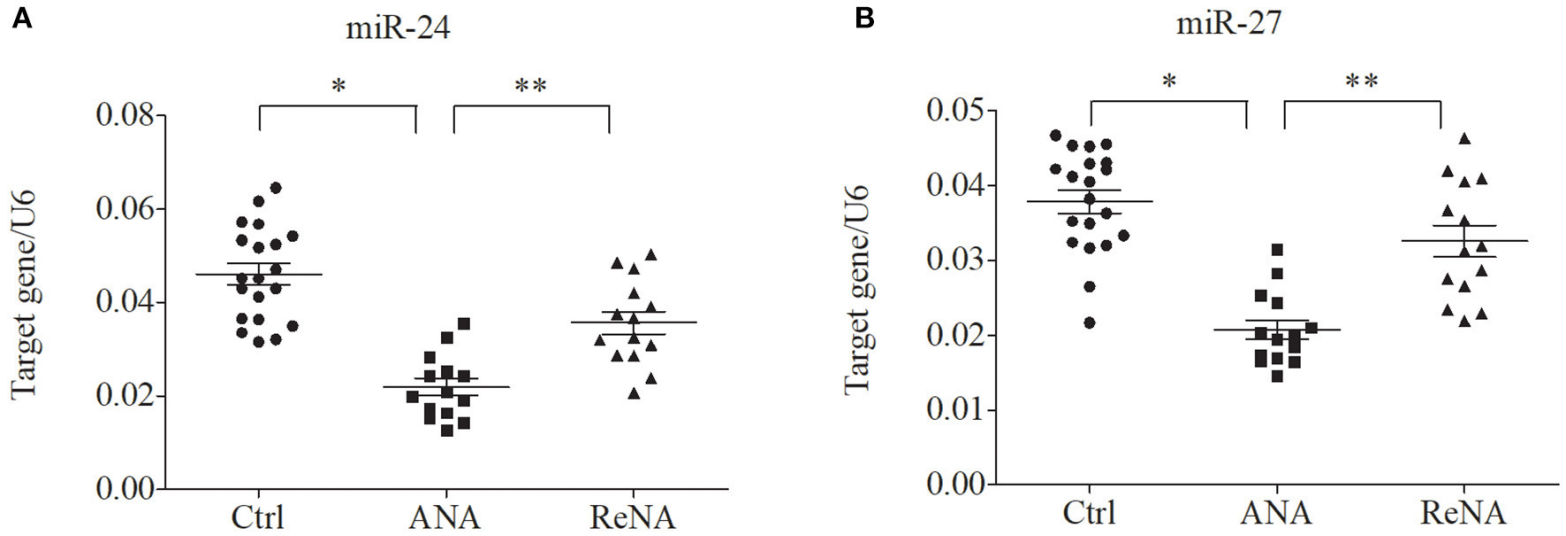
Children with INS have reduced expression of miR-24 and miR-27 in CD4^+^CD25^−^ T cells. Expression of miRNAs (relative to U6) were determined by real-time PCR. **(A)** Expression levels of miR-24. **(B)** Expression levels of miR-27. ^*^P < 0.05 and ^**^P > 0.05.

Previous research showed that miR-24 and miR-27 affected Th2 cell expression. Because we identified increased expression of Th2 cells in the ANA group, we also determined whether these miRNAs were also decreased (Figure 3). The results showed that the levels of miR-24 and miR-27 were significantly lower in the ANA group than in the Ctrl group (both *P* < 0.05; Table 1). The levels of these two miRNAs were greater in the ReNA group than in the ANA group, but not up to the levels in the Ctrl group (*P* < 0.05).

**Table 1:** Expression of miR-24 and miR-27 in patients with non-atopic INS and healthy controls.

	**Ctrl (*n* = 20)**	**ANA (*n* = 14)**	**ReNA (*n* = 14)**
miR-24	(46.03 ± 10.08) × 10^−3^	(21.84 ± 6.86) × 10^−3^^a^	(35.59 ± 9.10) × 10^−3^^b^
miR-27	(37.83 ± 6.83) × 10^−3^	(20.72 ± 4.93) × 10^−3^^a^	(32.55 ± 7.86) × 10^−3^^b^

*Values are expressed as mean ± SD*.

a *P < 0.05 for one-way ANOVA vs. Ctrl group*.

b *P < 0.05 for one-way ANOVA vs. ANA group*.

*Ctrl, healthy control; ANA, active phase non-atopic; ReNA, remission phase non-atopic*.

### miR-24 and miR-27 Suppress the Expression of Th2 Cells and IL-4

**Figure 4:**
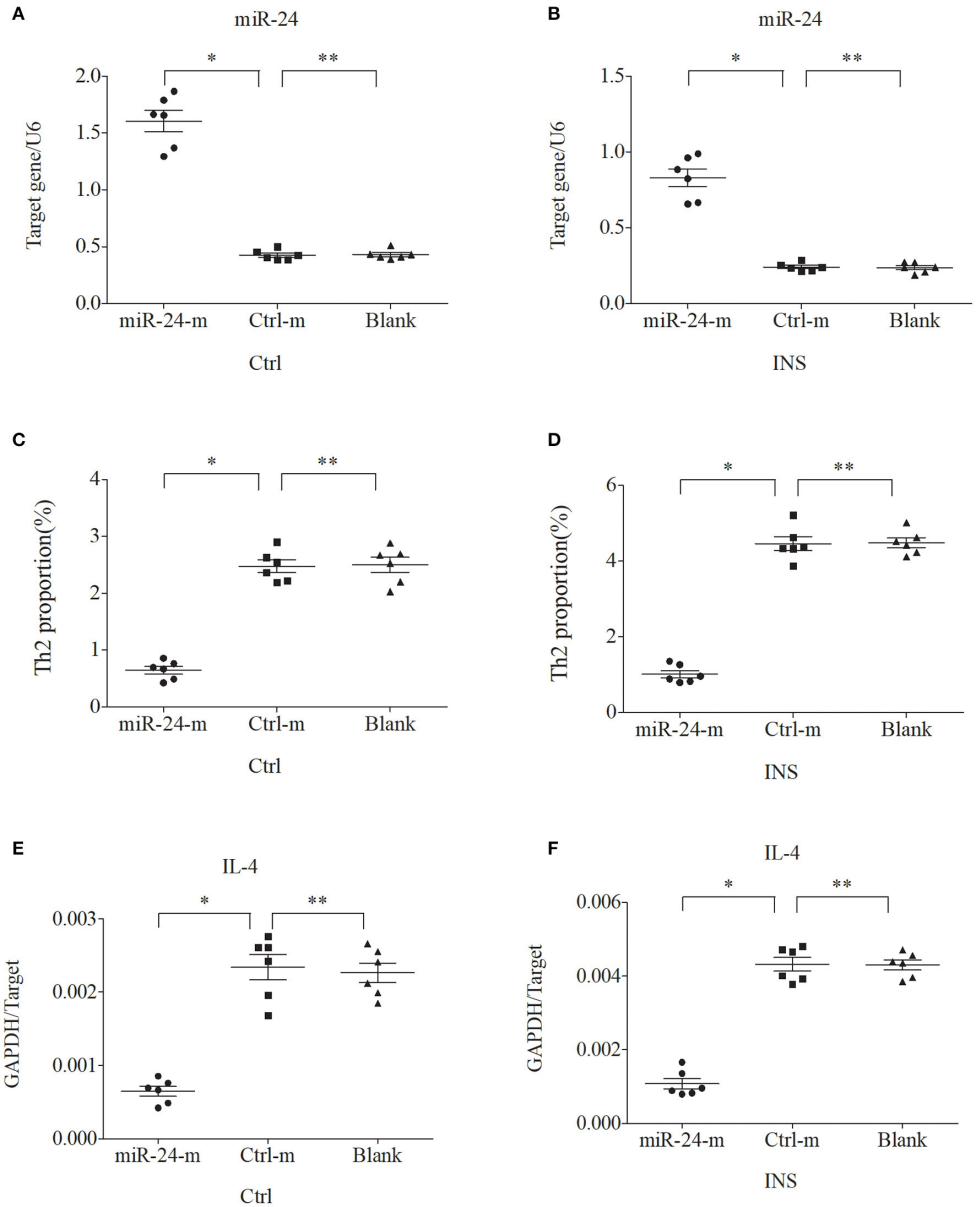
Up-regulation of miR-24 in PBMCs from controls and INS patients **(A,B)** reduces the percentage of Th2 cells **(C,D)** and the expression of IL-4 mRNA **(E,F)**. Here and in Figures 5–7: expression of miRNAs (relative to U6) and IL-4 (relative to GAPDH) were determined by real-time PCR; ANA (*n* = 6), Crtl (*n* = 6); and abbreviations are Ctrl-m (miRNA negative control), miR-24-m/miR-27-m (miR-24/miR-27 mimic), Ctrl-i (miRNA inhibitor [negative control]), and miR-24-i/miR-27-i (miR-24/miR-27 inhibitor). ^*^P < 0.05 and ^**^P > 0.05.

**Figure 5:**
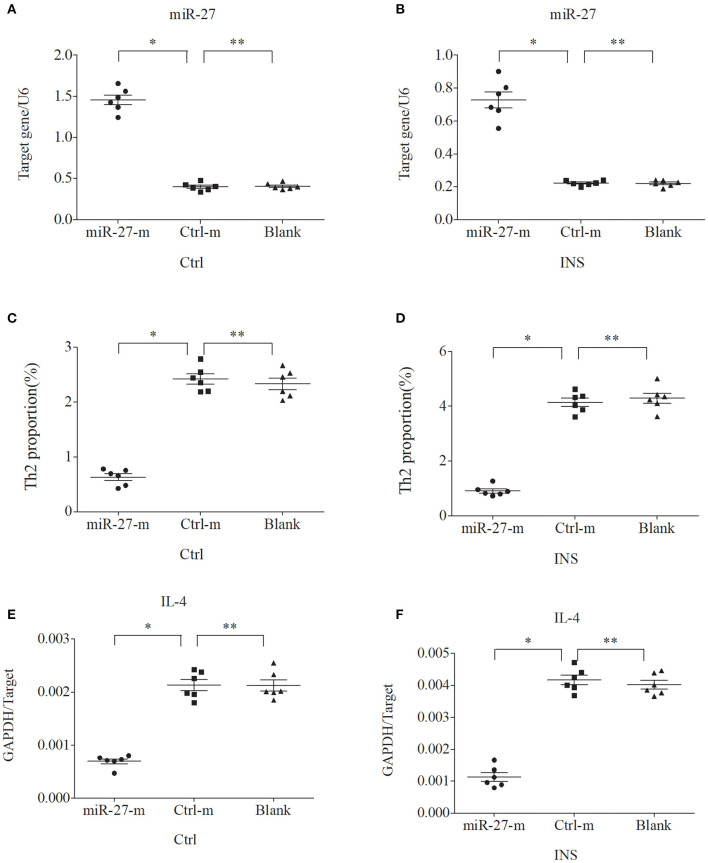
Up-regulation of miR-27 in PBMCs from controls and INS patients **(A,B)** reduces the percentage of Th2 cells **(C,D)** and the expression of IL-4 mRNA **(E,F)**. ^*^P < 0.05 and ^**^P > 0.05.

**Figure 6:**
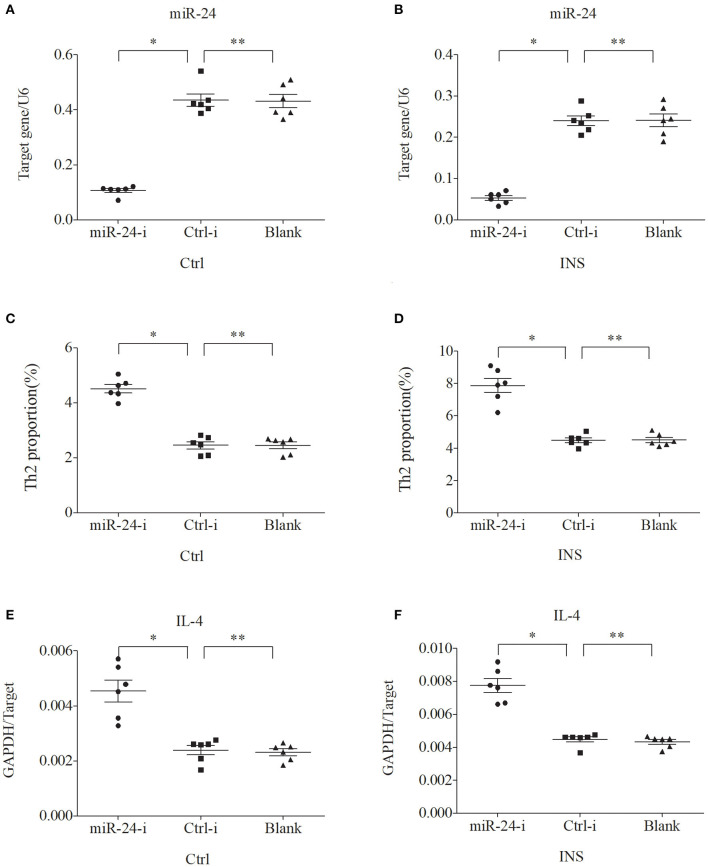
Down regulation of miR-24 in PBMCs from controls and INS patients **(A,B)** increases the percentage of Th2 cells **(C,D)** and the expression of IL-4 mRNA **(E,F)**. ^*^P < 0.05 and ^**^P > 0.05.

**Figure 7:**
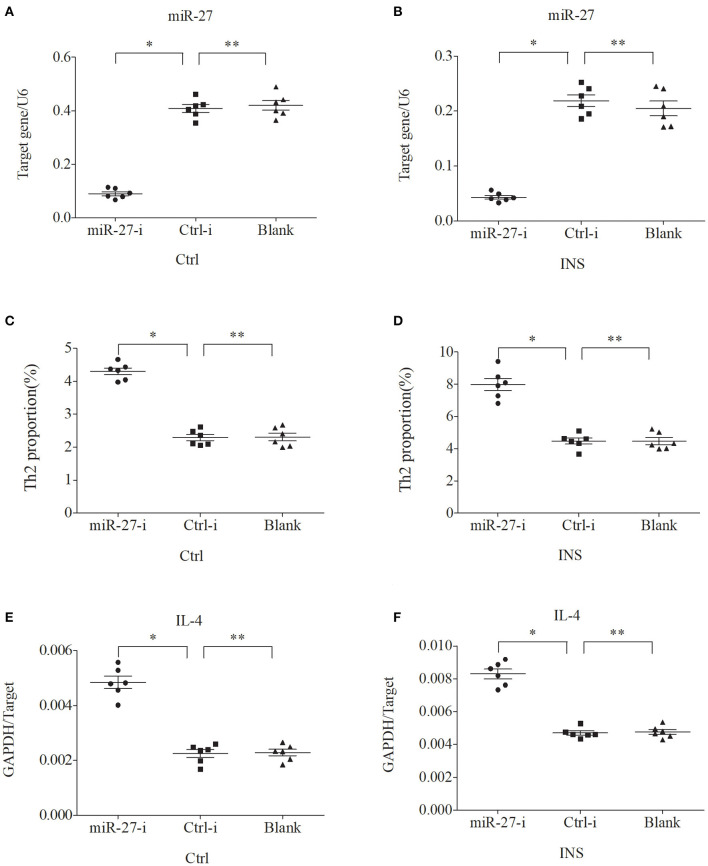
Down regulation of miR-27 in PBMCs from controls and INS patients **(A,B)** increases the percentage of Th2 cells **(C,D)** and the expression of IL-4 mRNA **(E,F)**. ^*^P < 0.05 and ^**^P > 0.05.

We further examined the relationship between miR-24, miR-27, and Th2 cells by transfection of PBMCs isolated from healthy volunteers and INS patients with ANA using six different miRNAs: negative control (Ctrl-m); miR-24 mimic (miR-24-m); miR-27 mimic (miR-27-m); miRNA inhibitor negative control (Ctrl-i); miR-24 inhibitor (miR-24-i); or miR-27 inhibitor (miR-27-i). The results showed that up-regulation of miR-24 (Figures 4A,B) and miR-27 (Figures 5A,B) reduced the percentage of Th2 cells (Figures 4C,D, 5C,D) and the expression of IL-4 mRNA (Figures 4E,F, 5E,F). In agreement, down regulation of miR-24 (Figures 6A,B) and miR-27 (Figures 7A,B), increased the percentage of Th2 cells (Figures 6C,D, 7C,D) and the expression of IL-4 mRNA (Figures 6E,F, 7E,F).

## Discussion

A large body of evidence has demonstrated that the immune system may play a crucial role in INS (3), although the pathogenic details of this disease remain mostly unknown. Previous research suggested that INS may be due to an abnormal T cell response or the dysregulation of T lymphocytes (3–8). MCD is the most common cause of INS (1, 2, 18, 19), and Th2 cells play an important role in many allergic and inflammatory diseases (9–11, 27, 31). More than 60 years ago, Hardwicke et al. reported an association of seasonal proteinuria with pollen sensitivity (12). Patients with MCD often have allergy-like symptoms, such as bronchial asthma, allergic rhinitis, atopic dermatitis, and urticaria. Some studies also confirmed that patients with INS may have increased levels of serum IgE, especially children with INS recurrence (13–15, 18). However, the causal relationship between the increased serum level of IgE or atopy and the pathogenesis of INS remains uncertain. The present study confirms that children in the active phase of INS with or without atopy had increased plasma levels of IgE compared with healthy controls, and they also had higher levels than children in the remission phase of INS. Among children in remission, the IgE levels of non-atopic children were nearly normal, but the IgE levels of atopic children were high. Thus, children with atopic and non-atopic INS have increased serum levels of IgE. However, further investigations of the underlying cytokine regulatory network are needed to more completely understand the relationship between INS and IgE production.

Th2 cells secrete IL-13 and IL-4, and this can promote the production of IgE in B cells (9–11). In particular, IL-13 induces a change from expression of IgM to IgE in B cells, and also induces podocytes to increase their synthesis of CD80. Increased CD80 expression by podocytes is associated with proteinuria (13–15, 18). Elevated levels of IL-13 are present in the urine and serum of patients who have kidney disease, and are also associated with a proteinuria (13–15, 18). These previous studies thus suggest that IL-13 functions in the pathogenesis of kidney disease, but the mechanism responsible for the increased IL-13 level is still unclear. Recent studies reported that Th2-related factors, IL-13 and IL-4, have increased levels in patients with active INS (13–15, 18). Other studies noted that patients with INS have increased proportions of Th2 cells (8, 18, 21, 22), but it was uncertain whether this alteration contributed to the pathogenesis of INS. This led us to examine the expression of Th2 cells in peripheral blood and the plasma concentrations of IL-13 and IL-4 in patients with different INS disease states. Our results demonstrated significantly increased levels of Th2 cells, IL-13, and IL-4 in patients with active phase INS with or without atopy. These results thus suggest that the proteinuria and increased IgE levels that occur during the active phase of INS, regardless of the presence of atopy, might be due to the increased levels of IL-13 and IL-4, which were caused by a drift toward Th2 cells. However, the mechanism responsible for this drift toward Th2 cells needs further study to elucidate the role of the immune system in the pathogenesis of INS.

miRNAs are subtle “master controllers” of gene expression, and function in the pathogenesis of many human diseases, especially chronic and multifactorial diseases (23, 24). Previous studies reported that some specific miRNAs were abnormally expressed in patients with various allergic and autoimmune diseases, such as asthma, systemic lupus erythematosus (SLE), and lupus nephritis, and that their expression also increased with disease activity (27, 32, 33). Other specific miRNAs are important for Th2 cell proliferation, differentiation, and immune function (27–29). In particular, miR-24 and miR-27 suppress allergic inflammation and target a network of regulators of Th2 cell-associated cytokine production (28, 29). Thus, the abnormal increase of Th2 cells and its related factor IL-13 in children with INS is not related to atopy, but is related to the pathogenesis of nephropathy. However, it is unclear whether alterations in the levels of different miRNAs lead to alterations in Th2 expression in patients with active INS. Therefore, we determined the expression of miR-24 and miR-27 and examined their possible role in regulating Th2 cell differentiation. Our results clearly showed that these two miRNAs had low expression in pediatric patients with active non-atopic INS. This suggests that the drift toward Th2 cells might be related to the low expression of these miRNAs in these children.

To verify this hypothesis, we first isolated PBMCs from healthy controls and children with non-atopic INS. Then, we transfected these cells with miR-24 or miR-27 mimics to increase expression, or with miR-24 or miR-27 inhibitors to reduce expression. The results showed that the percentage of Th2 cells and the level of IL-4 decreased when the levels of these miRNAs was increased. In agreement, the percentage of Th2 cells and the expression of IL-4 increased when the levels of these miRNAs was decreased. These results support our initial hypothesis that miR-24 and miR-27 reduce the percentage of Th2 cells, and suggest they might play an important role in Th2 expression during active INS.

In conclusion, our results indicate that the Th2 related-cytokine IL-13 is related to the pathogenesis of kidney disease but is not related to atopy, although the mechanisms responsible for the increased level of this cytokine remain unclear. Our results also indicate that pediatric patients with active phase INS that is either atopic or non-atopic have higher levels of IgE, and this might be related to their higher levels of IL-13 and IL-4 due to a drift toward Th2 cells. Children with active phase non-atopic INS had reduced levels of miR-24 and miR-27, an increased percentage of Th2 cells, and an increased level of IL-4, and this led to IL-13 over-expression and increased IgE levels. In other words, these two miRNAs suppress the expression of Th2 cells and play an important role in the drift toward Th2 cells in children with active INS. Therefore, we speculate that these miRNAs should be considered as potential targets for treatment of kidney disease. Our research thus provides a new research direction and suggests a possible novel treatment for INS.

## Data Availability Statement

The original contributions presented in the study are included in the article/supplementary materials, further inquiries can be directed to the corresponding author/s.

## Ethics Statement

The studies involving human participants were reviewed and approved by Ethics Committee of Shenzhen Children's Hospital. Written informed consent to participate in this study was provided by the participants' legal guardian/next of kin.

## Author Contributions

All authors listed have made a substantial, direct and intellectual contribution to the work, and approved it for publication.

## Conflict of Interest

The authors declare that the research was conducted in the absence of any commercial or financial relationships that could be construed as a potential conflict of interest.
